# Editorial for the Special Issue “Antimicrobial Resistance and Genetic Elements in Bacteria”

**DOI:** 10.3390/microorganisms11030670

**Published:** 2023-03-06

**Authors:** Maria Scrascia, Carla Calia, Carlo Pazzani

**Affiliations:** Department of Bioscience, Biotechnologies and Environment, University of Bari, Via Orabona 4, 70126 Bari, Italy

Throughout human history, bacterial infections have been an omnipresent threat, which have, on occasion, resulted in devastating pandemics affecting humanity [1]. We need to consider the fact that before the 1940s, bacterial infections were the most common cause of human death, with an average life expectancy at birth of 47 years [2]. The discovery of penicillin by Sir Alexander Fleming in 1928 led to high expectations and marked the beginning of the antibiotic era. The subsequent finding of new antibiotic molecules between 1950s and 1970s led researchers to believe that antibiotics would bring an end to these infections [3], a hope which was soon to be dashed by the discovery of antibiotic-resistant bacteria (ARB). The emergence and spread of ARB, mainly due to the overuse and inappropriate usage of antibiotics in clinical fields and, above all, in anthropic applications (e.g., zootechny), are now recognised as marking one of the major public health concerns worldwide [4,5,6]. It is forecasted that by 2050, bacterial infections could become the leading cause of death for human beings, a prediction supported by a recent study covering over 204 countries and territories, published in *The Lancet* journal in 2022 [7].

Antimicrobial resistance is mainly mediated by specific genes that are often harboured by genetic elements such as plasmids, integrons, transposons, insertion sequences, integrative conjugative elements, etc. [8]. Among such elements, plasmids (if conjugative or mobilisable) play key roles in the horizontal transfer of antimicrobial resistance genes (ARG) and in the emergence of new ARB [9,10]. A non-secondary role is also played by insertion sequences (e.g., IS26 and IS257) and transposons (e.g., Tn21), which can shape plasmids by the embedding and/or reassortment of ARG, with the final outcome of expanding the range and enrichment of ARG in plasmids [11,12,13].

This Special Issue, entitled “Antimicrobial Resistance and Genetic Elements in Bacteria”, aimed to collect new data on ARG and their dissemination through mobile or mobilisable genetic elements in both Gram-negative and Gram-positive bacteria. It comprises a review and eleven articles.

In addition to being a public health threat, zoonotic pathogens are responsible for major economic losses. The review published in this Special Issue offers a valuable update on ARG and their co-occurrence and genome localisation (mainly on integrative conjugative and integrative mobile genetic elements) in *Streptococcus suis*, a very important swine pathogen, which is also able to infect humans [14]. Animals, through their commonly harboured bacteria (e.g., intestinal tract), may act as a reservoir for both ARG and mobile genetic elements (MGE) involved in ARG horizontal transfer. The study on multidrug-resistant *Enterococcus faecalis* and *Enterococcus faecium* strains isolated from poultry clearly highlights such a role [15]. In the clinical field, antibiotic resistance is a matter of great relevance, since multidrug-resistant bacteria (MDR) are increasingly isolated from cases of both common and, above all, hospital-acquired infections. Of particular concern is the emerging spread of resistance to carbapenems and cephalosporins (e.g., those of third-generation), which are considered frontline antibiotics. *Klebsiella pneumoniae* and *Pseudomonas aeruginosa* are among the antimicrobial-resistant ESKAPE pathogens (*Enterococcus faecium*, *Staphylococcus aureus*, *Klebsiella pneumoniae*, *Acinetobacter baumannii*, *Pseudomonas aeruginosa*, and *Enterobacter* species) listed by the World Health Organization (WHO) as imminent threats to human health [16]. Two papers report on the characterisation of MDR *K*. *pneumoniae* strains isolated in Egypt and harbouring carbapenemase and extended spectrum β-lactamases genes localised on plasmids. These studies highlight the widespread diffusion of similar plasmids and their roles in conferring multidrug resistance [17,18]. Similarly, another study published in this Special Issue provides data on carbapenemase and extended β-lactamase genes harboured by a plasmid conferring MDR in *P*. *aeruginosa* [19]. From a clinical point of view, the widespread isolation of MDR strains resistant to carbapenems and cephalosporins has greatly limited the range of antibiotics which can be used to effectively treat infectious diseases (caused by Gram-negative bacteria), leaving colistin as an antibiotic last resort. Even the value of colistin has been undermined in the last decade by the discovery of colistin-resistant bacteria caused by the acquisition of plasmid-mediated colistin-resistant (*mcr*) genes [20,21,22]. Data on non-conjugative but mobilisable plasmids are constantly expanding, disclosing their important roles in both the horizontal spread of ARG and the insurgence of MDR bacteria. Current knowledge on plasmids shows how complex their world is, and how much remains to be learnt about it. For instance, studies on hybrid or mosaic plasmids are now starting to reveal the means by which plastic plasmids enable the emergence of new plasmids which have the acquired properties of virulence, antibiotic resistance, and/or the ability to be horizontally transferred (e.g., through the acquisition of *mob* genes and/or *oriT* sequences). This Special Issue offers examples of such studies [18,23]. It is worth noting the identification of a mosaic IncR plasmid that has acquired a region harbouring ARG from IncI1 plasmids and, more importantly, *oriT* and *nikAB*, enabling its mobilisation in the presence of helper conjugative plasmids [23]. This Special Issue also includes studies on the following topics: the relationship between antibiotic resistance and biofilm-related genes in *Pseudomonas* species isolates, drug resistance detected in *Cedecea neteri* (a clinical opportunistic pathogen), the mobilisation of kanamycin-resistant Col-like plasmids, and the horizontal exchange of a MDR IncFII plasmid between *E*. *coli* and *K*. *quasipneumoniae*, which occurred in the same patient [24,25,26,27].
